# *Achillea schurii* Flowers: Chemical, Antioxidant, and Antimicrobial Investigations

**DOI:** 10.3390/molecules21081050

**Published:** 2016-08-12

**Authors:** Daniela Benedec, Daniela Hanganu, Ilioara Oniga, Lorena Filip, Cristina Bischin, Radu Silaghi-Dumitrescu, Brînduşa Tiperciuc, Laurian Vlase

**Affiliations:** 1 Department of Pharmacognosy, “Iuliu Haţieganu” University of Medicine and Pharmacy, 12 I. Creangă Street, Cluj-Napoca 400010, Romania; dbenedec@umfcluj.ro (D.B.); ioniga@umfcluj.ro (I.O.); 2 Department of Bromatology, Hygiene, Nutrition, “Iuliu Haţieganu” University of Medicine and Pharmacy, 6 Pasteur Street, Cluj-Napoca 400349, Romania; 3 Department of Chemistry and Chemical Engineering, “Babeş-Bolyai” University, 11 A. Janos Street, Cluj-Napoca 400028, Romania; cbischin@chem.ubbcluj.ro (C.B.); rsilaghi@chem.ubbcluj.ro (R.S.-D.); 4 Department of Pharmaceutical Chemistry, “Iuliu Hațieganu” University of Medicine and Pharmacy, 41 V. Babeş Street, Cluj-Napoca 400012, Romania; btiperciuc@umfcluj.ro; 5 Department of Pharmaceutical Technology and Biopharmaceutics, “Iuliu Haţieganu” University of Medicine and Pharmacy, 12 I. Creanga Street, Cluj-Napoca 400010, Romania; laurian.vlase@umfcluj.ro

**Keywords:** polyphenols, *Achillea schurii*, antioxidant, antimicrobial, hemoglobin, cytochrome c

## Abstract

This study aims to evaluate the phenolic profile, and antioxidant and antimicrobial activity of *Achillea schurii Sch.-Bip.*, an endemic species from Romania that has not been investigated yet. The chromatographic profile of the phenolic components was obtained using the HPLC-MS method, while the total polyphenol, flavonoid, caffeic acid derivative contents were quantified using spectrophotometric methods. The antioxidant activity was evaluated using different methods: DPPH radical scavenging, hemoglobin ascorbate peroxidase activity inhibition (HAPX), inhibition of lipid peroxidation catalyzed by cytochrome c, and direct detection of plant-derived free radicals using electron paramagnetic resonance (EPR). The antimicrobial test was performed using the disk diffusion assay. The phenolic profile has revealed high amounts of isoquercitrin, rutin, luteolin, and apigenin. The *A. schurii* extract exhibited a good antioxidant capacity, and high phenolic contents (76.93 mg/g polyphenols, 18.61 mg/g flavonoids and 41.48 mg/g caffeic acid derivatives, respectively). The antimicrobial tests reveal a remarkable inhibitory activity against *Listeria monocytogenes*, *Staphylococcus aureus*, and *Salmonella typhimurium*. Considering the above, *A. schurii* may be deemed to offer good perspectives for pharmaceutical and industrial applications.

## 1. Introduction

*Achillea schurii Schultz Bip.* (synonyms: *A. atrata Baumg*, *A. oxyloba (DC.) Schultz Bip. ssp. schurii (Schultz Bip.) Heimerl, Anthemis schurii Schultz Bip.*) is an endemic species from the Eastern and Southern Carpathians. In Romania, this endemic species is a perennial, herbaceous, alpine-subalpine plant [1,2,3]. *A. schurii* and over 100 other species worldwide belong to the Achillea *L.* genus, respectively *Asteraceae* family [3,4,5,6]. Proazulenes (achillicin), essential oils, flavonoids, coumarins, terpenoids (monoterpenes, sesquiterpenes, diterpenes, triterpenes), sterols, lignans, amino acid derivatives, and alkamides have been found in *Achillea* species and they are involved in the high therapeutic value of these species [7,8,9,10,11,12,13,14,15,16,17,18,19,20]. Polyphenolic compounds, such as those present in *Achillea* species, are known for their potential protective role against oxidative stress, which causes coronary heart disease, stroke, diabetes, cancers, osteoporosis, neurodegenerative disease, etc. [21]. 

This species is less investigated and less used than other *Achillea* species (*A. millefolium*, *A. distans*, *A. collina*, *A. pannonica*, *A. biebersteinii*, etc.) [1,2,5,6]. The scientific information about this plant is very limited or does not exist at all. Scientific literature only offers taxonomic and systematic data.

The purpose of this work was to identify and quantitatively determine the phenolic compounds of the *Achillea schurii* flowers and to investigate their antioxidant and antimicrobial properties. This study contributes to an increase in knowledge about this species, and also raises awareness on the potential therapeutic uses for obtaining various therapeutic alternatives, which may lead to better health care in the near future.

## 2. Results and Discussion

### 2.1. HPLC Analysis of Polyphenolic Compounds

**Figure 1:**
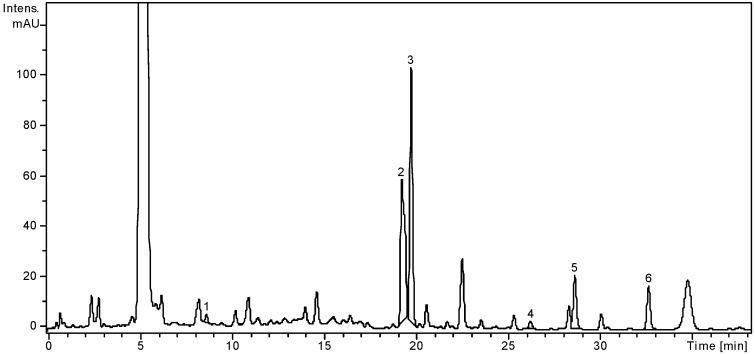
HPLC chromatogram of *A. schurii* extract. Notes: Chromatographic conditions were as given in the Experimental Section. The identified compounds: 1, *p*-coumaric acid; 2, isoquercitrin; 3, rutin; 4, quercetin; 5, luteolin; 6, apigenin.

A method of coupling HPLC with MS was optimized for the separation and identification of phenolic acids and flavonoids [8,9,22,23,24,25,26,27]. This is the first report related to the analysis of the phenolic composition of *A. schurii* flowers. In this study, 19 standard phenolic compounds were employed: 8 phenolic acids and 11 flavonoids. The simultaneous analysis of different classes of polyphenols was performed by single column pass, and the separation of all examined compounds was carried out in 35 min [8,9,22,23,24,25,26,27]. Using this method, nine of them were identified for the first time in this work: four phenolic acids (gentisic, caffeic, chlorogenic, *p*-coumaric acids) and five flavonoids (isoquercitrin, rutin, quercetin, apigenin, and luteolin). Considering the 19 standard compounds used in this study, some other peaks were not identified. The concentrations of the polyphenolic compounds found in the analyzed sample are shown in Table 1. The HPLC chromatogram of the *A. schurii* sample is shown in Figure 1.

These results indicated the highest levels of flavonoid glycosides: isoquercitrin (68.75 mg/100 g) and rutin (76.58 mg/100 g). Three phenolic acids—gentisic, caffeic, chlorogenic acids—were found in too low concentrations to be quantified (<0.02). The *p*-coumaric acid, an antioxidant predominant in yarrow (*A. millefolium*, *A. distans*, *A. biserratae*, *A. beibrestinii*, etc.), was found in the *A. schurii* extract with a concentration of 0.76 mg/100 g [8,9,17,28]. Several flavonoid aglycones—quercetin (1.05 mg/100 g), luteolin (8.73 mg/100 g), and apigenin (10.04 mg/100 g)—were found and quantified in this plant extract. These phenolic compounds having important therapeutic properties (e.g., anti-inflammatory, antibacterial, antiviral, antioxidant, neurotrophic, anti-tumor effects) were found to be the main flavonoids in several species of *Achillea*: *A. distans*, *A. ligustica*, *A. collina*, *A. millefolium*, etc. [8,9,19,29,30,31,32,33]. We may thus conclude that *A. schurii* may also be considered an important source of bioflavonoids, with significant therapeutic potential. Therefore, further studies are required to prove the therapeutic actions related to the presence of these compounds.

### 2.2. Total Polyphenol, Flavonoid, and Caffeic Acid Derivatives Content and Antioxidant Activity

The total polyphenol content (TPC) was expressed in gallic acid equivalents (mg GAE/g plant material), the total flavonoid content was expressed in rutin equivalents (mg RE/g plant material), and the total caffeic acid derivatives content was expressed in caffeic acid equivalents (mg CAE/g plant material) [9,27,34,35,36,37]. The results are shown in Table 2. 

**Figure 2:**
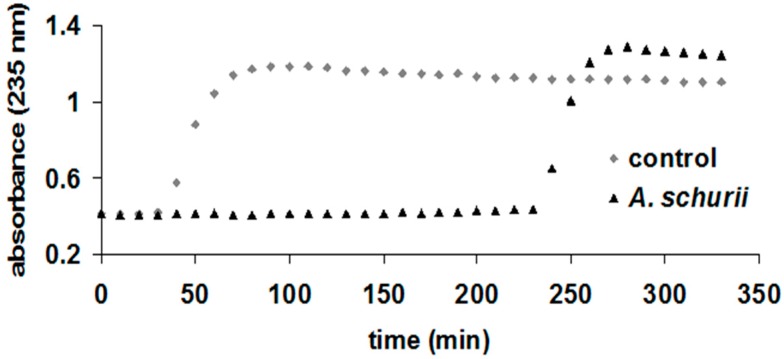
Liposome oxidation by cytochrome c in the presence of *A. schurii* extracts diluted in phosphate buffer, 10 mM, pH 7.

*A. schurii* thus contains comparable amounts of TPC (96.93 mg/g) and caffeic acid derivatives (41.48 mg/g) with other species of the *Achillea* genus. For *A. distans* subsp. *distans*, the contents of TPC and flavonoids were established at 101.61 mg GAE/g, and 37.26 mg RE/g, respectively, while for *A. millefolium*, the contents were established at 123.9 mg GAE/g, and 41.2 mg QE/g, respectively [9,16]. In this regard, the ethanolic extract of *A. schurii* was screened for its antioxidant activity using four in vitro assay models: DPPH bleaching, hemoglobin ascorbate peroxidase activity inhibition (HAPX), inhibition of lipid peroxidation catalyzed by cytochrome c, and electron paramagnetic resonance (EPR) spectroscopy [9,24,27,38,39,40,41,42,43]. Until now, there have been no scientific data on the antioxidant capacity of this species. The results obtained by DPPH bleaching assay are shown in Table 2 above. The IC_50_ value of the extract was 58.87 µg/mL. The ethanolic extract of *A. schurii* flowers exhibited higher antioxidant capacity (IC_50_ around 50 µg/mL) than *A. distans* subsp. *distans* (IC_50_ = 204.83 µg/mL) and *A. distans* subsp. *alpina* (IC_50_ = 83.80 µg/mL) [9]. Very good antioxidant capacity was also obtained with HAPX, and excellent results were recorded using the inhibition of liposomes oxidation by cytochrome c assay, as well. These methods involve the interaction of two metalloproteins found in the body—hemoglobin and cytochrome c, respectively—with the strong but biologically-relevant oxidant agent hydrogen peroxide in the presence of the antioxidants found in the sample. It is well known that the interaction of hemoglobin and cytochrome c leads to the formation of high-valent iron species, which will typically decay by abstracting electrons from the neighboring molecules in processes that occur under physiological conditions in the human body and are accelerated under certain pathologies. Thus, both methods monitor the capacity of antioxidants (polyphenols or phenolic acids in this case) to inhibit the formation of physiologically-relevant protein-based ferryl species [40,42,43]. The results obtained for HAPX were reflected in mg RE/g plant (Table 2), while those for the cytochrome c experiment were expressed in the delay of lipid oxidation (200 min) (Figure 2).

It may be assumed that the polyphenols show antioxidant activity via an iron chelating mechanism, because the flavonoids of the *A. schurii* extract (quercetin, isoquercetrin, rutin, etc.) have one catechol group in one of the aromatic rings and two hydroxy groups in the o-position that are essential for the chelation of iron [44].

According to the results obtained from the HAPX assay, the *A. schurii* extract has exhibited better capacity to protect hemoglobin against peroxide than that observed for *A. millefolium* (1000 vs. 724 mg RE/g), *A. collina*, *A. stricta*, *A. nobilis* (unpublished data). In the liposome experiments, *A. schurii* has had better antioxidant capacity (as manifested in a longer induction time for the onset of UV-detectable lipid oxidation) than that observed for *A. stricta* (44 min) and *A. nobilis* (100 min) but lower than the capacity for *A. distans* (500 min) or *A. millefolium*. These results are in line with the TPC and flavonoid content. Perhaps of note, for the liposome experiment, these extracts needed dilution by one order of magnitude more than for other previously examined plant extracts, such as *Lycium barbarum* [26] or *Hedera helix* extracts [43], in order for the induction time to be measurable in the same range.

### 2.3. Electron Paramagnetic Resonance (EPR) Detection of A. schurii Free Radicals

**Figure 3:**
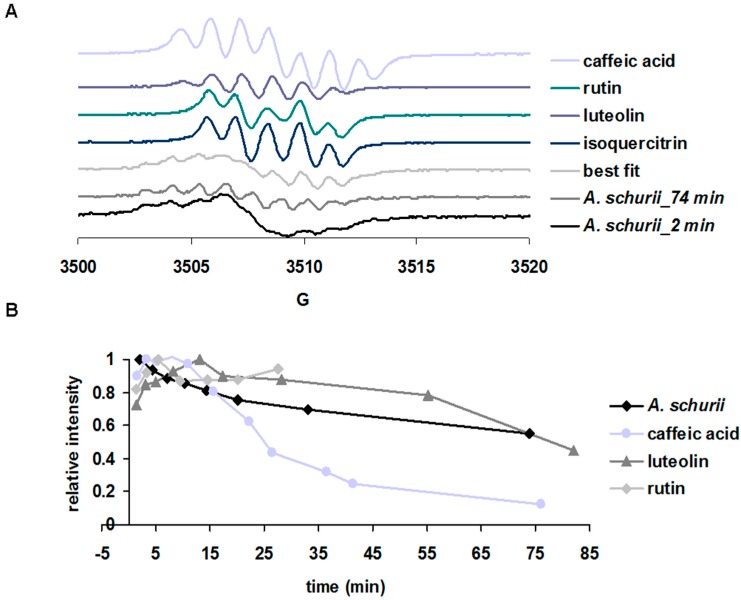
(**A**) The EPR spectra of the extract; (**B**) Radical decay kinetic curves of the 5 mM NaOH treated *A. schurii* extracts in 90% ethanol and of the 2 mM caffeic acid; luteolin and rutin shown as references run under the same conditions. Note: (**A**) The *A. schurii* extract was diluted 10 times and treated with NaOH, in ethanol 90%, recorded at the time indicated in the legend, alongside the spectra of 2 mM caffeic acid, rutin, luteolin, isoquercitrin, obtained under the same conditions (the spectra of the pure substances were scaled down by a factor of 2 compared to the natural extract, for clarity). The “best fit” spectrum was obtained as a weighted sum of 10% rutin, 10% luteolin, 10% isoquercetin, 50% caffeic acid, and 20% chlorogenic acid.

The initial EPR spectra generated as a result of auto-oxidation of the polyphenols under alkaline conditions from ethanolic extracts of *A. schurii* are shown in Figure 3A, along with the initial spectra from rutin, luteolin, isoquercitrin, and caffeic acid obtained as references under similar conditions. These signals were unstable in time (Figure 3A) (*A. schurii*, 60 min and B) and may be attributed to the formation of semiquinone anion radicals within the polyphenols. The B-ring in a flavonoid is the most important part in the molecule involved in both antioxidant and pro-oxidant reactivity. It is expected that within a complex natural extract, a huge variety of such radicals with different rates in formation and decay may be present [41,42]. The initial spectrum of the *A. schurii* extract, collected at ~2 min after exposure to alkaline pH, had a width of approximately 230 G, which is wider than the spectrum of isoquercitrin, rutin (155 G), and luteolin (185 G), i.e., of the major polyphenolic components. This spectrum also has an incompletely defined shape, most likely due to overlapping components from several polyphenolic free radicals. However, the spectrum collected on the same extract approximately one hour after the exposure to alkaline pH (Figure 3A) was much better defined in shape and can be simulated as a sum of individual spectra as shown in Figure 3A. In this “best fit” simulation, the highest contribution was due to the caffeic acid (50%), followed by chlorogenic acid (20%) and polyphenols (rutin, luteolin, and isoquercitrin–10%). Thus, even if the concentration of polyphenolic acids in the *A. schurii* extract was low (cf. Table 1, there was distinctly more rutin and isoquercitrin than chlorogenic or caffeic acid) and they can generate free radicals that can be detected by EPR and even dominate the lineshape. The decay kinetics of the signal (Figure 3B) provides further insight: they match to some extent the kinetics of luteolin (the fourth most abundant component according to the HPLC-MS data), but less those of rutin (the most abundant antioxidant in the sample cf. HPLC-MS) or caffeic acid (the dominant signal according to the lineshape fitting). The relative contributions of the various free radicals to the overall EPR signal of the natural extract should then not simply and directly correlate with the relative concentrations of the respective chemicals, but should be also marked by the relative reactivities/stabilities of these radicals. Overall, the EPR methodology described here offers a way to explicitly probe the chemical composition of a natural extract [41,42], in manners complementary to other methods.

### 2.4. In Vitro Antimicrobial Activity

The ethanolic extract was investigated for its antimicrobial properties against four bacteria species and one fungus (Table 3). The in vitro antimicrobial activity was performed by the agar disc diffusion method [9,45,46]. This is the first report that provides data on the antimicrobial potential of the *A. schurii* flowers extract.

Antimicrobial activity results were shown in Table 3. A one-way ANOVA test applied on the values in this table has revealed that the difference between the *Achillea* extract and the positive controls was statistically different for all microbial strains (0.001 < *p* < 0.05). Thus, the *A. schurii* extract was found to be inactive on the *C. albicans* (*p* ˂ 0.001). There was a slight activity against *E. coli*, and moderate antibacterial activities against *S. aureus* and *S. typhimurium* (*p* ˂ 0.001). The best results were observed against *L. monocytogenes*, the strong effect being illustrated by the 22 mm inhibition diameter—even more potent than the Gentamicin used as a reference antibiotic (0.001 < *p* < 0.05). The extract showed better antibacterial activity than *A. distans* on all tested bacteria [9]. Therefore, the extract of *A. schurii* may be an alternative for the control of *L. monocytogenes* in the food industry and a potential antibacterial agent. 

## 3. Experiment Section

### 3.1. Plant Material 

The flowers of *A. schurii* (Voucher No. 955) were identified and harvested in 2014 from the spontaneous flora of the Romanian Carpathians (Prahova County, Bucegi Mountains, East of Cabana Babele, Sinaia, Romania), during the flowering period, by D.H. 

### 3.2. Chemicals 

The phenolic acids (chlorogenic, caffeic acid, *p*-coumaric acids) and the flavonoids (rutin, isoquercitrin, quercitrin, hyperoside, myricetol, fisetin, quercetin, apigenin, kaempferol) were acquired from Sigma (St. Louis, MO, USA). The ferulic acid, sinapic acid, gentisic acid, gallic acid, patuletin, and luteolin were from Roth (Karlsruhe, Germany), the cichoric acid, caftaric acid were from Dalton (Toronto, ON, Canada). The sodium molybdate dihydrate, sodium nitrite, sodium hydroxide, sodium carbonate, hydrogen peroxide, sodium ascorbate, and bovine hemoglobin were purchased from Sigma-Aldrich (Steinheim, Germany). The HPLC grade methanol, analytical grade orthophosphoric acid, hydrochloric acid, aluminum chloride, sodium acetate, ethanol and Folin-Ciocalteu reagent were purchased from Merck (Darmstadt, Germany). The DPPH (2,2-diphenyl-1-picrylhydrazyl) was obtained from Alfa-Aesar (Karlsruhe, Germany). All microorganism products were distributed by MicroBioLogics^®^ (St. Cloud, Minnesota USA 56303): *Staphylococcus aureus* ATCC 49444 (Gram-positive bacteria), *Listeria monocytogenes* ATCC 13076 (Gram-positive bacteria), *Escherichia coli* ATCC 25922 (Gram-negative bacteria), *Salmonella typhimurium* ATCC 14028 (Gram-negative bacteria), and one fungal strain, *Candida albicans* ATCC10231. All spectrophotometric data were acquired using a Jasco V-530 UV-vis spectrophotometer (Jasco International Co., Ltd., Tokyo, Japan).

### 3.3. Preparation of the Sample Solution

The plant material was reduced to a proper degree of fineness. Five grams of powder were extracted with 50 mL of 70% ethanol (Merck, Darmstadt, Germany), for 30 min on a water bath, at 60 °C. The sample was then cooled down and centrifuged at 4500 rpm for 15 min, and the supernatant was recovered [9,24,27].

### 3.4. HPLC Analysis of the A. schurii Extract 

#### 3.4.1. Apparatus and Chromatographic Conditions for the Analysis of Polyphenolic Compounds

HPLC-MS analysis was performed using the chromatographic conditions previously described [8,9,22,23,24,25,26,27]. The determination of polyphenolic compounds was made using an Agilent 1100 HPLC Series system (Agilent, Santa Clara, CA, USA) equipped with G1322A degasser, G13311A binary gradient pump, column thermostat, G1313A autosampler, and G1316A UV detector that was coupled to an Agilent 1100 mass spectrometer (Agilent). The separation of the phenolic components was achieved on a reverse-phase analytical column (Zorbax SB-C18 100 × 3.0 mm i.d., 3.5 μm particle). The detection was made on both UV (λ = 330 nm until 17.5 min, then at λ = 370 nm) and MS system using an electrospray ion source in negative mode. The chromatographic data were processed using ChemStation and DataAnalysis software (Agilent, version B01.03, Palo Alto, CA, SUA) from Agilent. The prepared mobile phase with methanol and acetic acid 0.1% (*v*/*v*) was used in a binary gradient. For 35 min, the elution had been linear gradient, starting at 5% methanol and finishing at 42% methanol (maintained for 3 min). The flow rate of mobile phase was 1 mL·min^−1^. Five microliters were used for injection. For the qualitative analysis, MS signal only was used. The standard MS spectra were integrated in a mass spectra (Bruker Daltonics GmbH, version 5.3, Bremen, Germany) library. In these chromatographic conditions, the couples—caftaric acid with gentisic acid, and caffeic acid with chlorogenic acid—could not be quantitatively determined due to overlapping. The polyphenolic compounds of the *A. schurii* extract were identified based on their retention times, UV and MS spectra as compared to the standards. The quantification of compounds was performed using an external standard method [8,9,22,23,24,25,26,27].

#### 3.4.2 Analysis of Phenolic Compounds

The detection and quantification of polyphenols were performed in UV completed by mass spectrometry detection [8,9,22,23,24,25,26,27]. Due to peak overlapping, four polyphenol-carboxylic acids (caftaric, gentisic, caffeic, chlorogenic acids) were determined only based on MS spectra, whereas for the rest of the compounds, the linearity of the calibration curves was very good (R^2^ > 0.998), with detection limits in the range of 18 to 92 µg/mL The detection limits were calculated as the minimal concentration yielding a reproducible peak with a signal-to-noise ratio greater than three. Quantitative determinations were performed using an external standard method; retention times were determined with a standard deviation ranging from 0.04 min to 0.19 min. For all compounds, the accuracy was between 94.13% and 105.3%. In the sample, the compounds were identified by comparison of their retention times and recorded electrospray mass spectra with those of standards in the same chromatographic conditions.

### 3.5. Determination of Polyphenols Content

#### 3.5.1. Determination of the Total Polyphenolic Content

The total polyphenolic content of the *A. schurii* extract was determined using the protocol described in the European Pharmacopoeia, using the Folin-Ciocalteu reagent, with a calibration curve of gallic acid (R^2^ = 0.999) [9,27,34,35,36]. Two milliliters of ethanolic extract was diluted 25 times, than mixed with 1.0 mL of Folin-Ciocalteu reagent, 10.0 mL of distilled water and diluted to 25.0 mL with a 290 g/L solution of sodium carbonate. The sample was incubated in the dark for 30 min. The absorbance was measured at 760 nm using a UV-VIS Jasco V-530 spectrophotometer (Jasco International Co., Ltd., Tokyo, Japan). The content of total phenols was expressed as mg of gallic acid equivalents extracted from 1.0 g of dried plant material.

#### 3.5.2. Determination of the Flavonoid Content

The spectrophotometric aluminum chloride method was used for the flavonoid determination [9,37]. Five milliliters of each extract were mixed with 5.0 mL of sodium acetate 100 g/L, 3.0 mL of aluminum chloride 25 g/L, and filled up to 25 mL with methanol in a calibrated flask. The absorbance was measured at 430 nm [36]. The total flavonoid content value, expressed as rutin equivalent (RE), was determined using a calibration curve based on rutin (R^2^ = 0.999). The results are expressed in rutin equivalents (mg RE/g dried plant material).

#### 3.5.3. Determination of the Caffeic Acid Derivatives Content

The caffeic acid derivatives were determined using a spectrometric method using Arnows’ reagent (10 g sodium nitrite and 10 g sodium molybdate made up to 100 mL with distilled water) as previously described in the Romanian Pharmacopoeia (10th Edition—*Cynarae folium* monograph) [9,27,37]. The percentage of phenolic acids, expressed as caffeic acid equivalent on dry material plant (mg CAE/g dried plant material), was determined using an equation that was obtained from the calibration curve of the caffeic acid (R^2^ = 0.994). 

### 3.6. Determination of Antioxidant Properties of the A. schurii Extract

#### 3.6.1. DPPH Bleaching Assay

For the DPPH assay, 2.0 mL of methanolic DPPH solution (0.25 mM) were added to 2.0 mL of extract solution (or standard) in ethanol at different concentrations (18.75–150 μg/mL). After 30 min of incubation at 40 °C in a thermostatic bath, the decrease in absorbance was measured at 517 nm. The percent of DPPH scavenging ability was calculated as: DPPH scavenging ability = (A_control_ − A_sample_/A_control_) × 100, where Acontrol is the absorbance of DPPH radical and methanol (containing all reagents except the sample) and Asample is the absorbance of DPPH radical and sample extract. The percentage of DPPH consumption was converted to quercetin equivalents using a calibration curve (R^2^ = 0.985) of quercetin standard solutions (0.5–5 μg/mL). Quercetin is one of the well-known antioxidants [47]. The IC_50_ value that means the concentration of sample required to scavenge 50% of DPPH free radicals was calculated [9,24,27,38,39].

#### 3.6.2. Hemoglobin/Ascorbate Peroxidase Activity Inhibition (HAPX) Assay

The hemoglobin ascorbate peroxidase activity assay (HAPX) was described in detail in [26,40,41,42]. The reaction was monitored at 405 nm, where all the changes are due to the hemoglobin transformation. Met hemoglobin (6 µM) was added to a mixture of ascorbate (120 μM), peroxide (700 μM) and extracts (5 μL), in acetate buffer, pH 5.5 to start the reaction. An increase in the inhibition time denotes a good antioxidant capacity of the tested extract which acts in competition with the ascorbate. The percentage of the inhibition time for each case was converted to rutin equivalents (RE) using a calibration curve (R^2^ = 0.980) with rutin standard solutions of 0–1.5 mM. 

#### 3.6.3. Inhibition of Lipid Peroxidation Catalyzed by Cytochrome c

5 mg/mL soybean lecithin were suspended in phosphate buffer (20 mM, pH 7) and sonicated for 15 min in an ultrasonic bath (using a Power Sonic 410 device Thermoline Scientific, Wetherill Park, NSW, Australia) to obtain liposomes. The liposome oxidation experiment was run at room temperature, for 350 min, in the presence of cytochrome c (2 µM) and extracts (diluted 16,000 times) by monitoring the absorbance at 235 nm, where the formation of lipid conjugated dienes can be observed [43].

#### 3.6.4. Free Radical Generation Experiment

For the EPR experiment, the extracts were diluted 10 times in 90% ethanol, followed by the treatment with 5 mM NaOH. A low quantity (100 µL) of sample was rapidly transferred to a glass capillary EPR tube. The capillary was placed in the holder of a Bruker ELEXSYS E-580 spectrometer (Bruker, Billerica, MA, USA) with continuous wave at X band (~9.4 GHz). The spectra were measured at room temperature with the following parameters: modulation frequency 100 kHz, microwave power 9.6 mW, modulation amplitude 0.5 G, center field 3514, and sweep field 100 G [42]. 

### 3.7. Determination of Antimicrobial Activity

The extract of *A. schurii* was investigated for activity against *Staphylococcus aureus*, *Listeria monocytogenes*, *Salmonella typhimurium*, *Escherichia coli*, and *Candida albicans*, using a disc-diffusion assay. After the hydration of the lyophilized strain, the sterile tampon was impregnated with hydrated material and transferred on the selective medium specific for each strain (e.g., *Salmonella*: Rambach agar, XLD agar; *E. coli*: TBX agar). The tampon was rotated with pressure and a circular area was inoculated on the agar media. Using a sterile loop, streaks were made repeatedly in the inoculated area and then streaked also on the rest of the plate’s surface. Immediately afterwards, the culture medium inoculated was incubated at corresponding temperatures (e.g., *Salmonella* 37 °C; *E. coli* 44 °C). From the pure ATCC reference culture, of 24 h, a 0.5 McFarland suspension was obtained (corresponding to 10^8^ CFU/mL). The Muller-Hinton agar plates were inoculated by inundation. The plates were then dried in the thermostat for 20 min (this interval is not exceeded because the bacteria might reach a multiplication phase). The sterile disks were soaked with the tested solutions. The plates were incubated overnight at 37 °C and the results were recorded by measuring the diameter of the inhibition zone (mm). Gentamicin and Fluconazole were used as standard drugs. The negative control was 70% ethanol. The clear halos greater than 10 mm were considered as positive results [9,24,45,46].

### 3.8. Statistical Analysis 

The samples have been analyzed in triplicate or more; the average and the relative SD have been calculated using the Excel software package. The experimental data have been evaluated using one-way analysis of variance (ANOVA), with *p* < 0.05 as threshold value for statistical significance. The statistical results confirm the hypothesis that the differences between the results are either not significant (*p* > 0.05), significant (0.001 < *p* < 0.05), or highly significant (*p* < 0.001). 

## 4. Conclusions

In summary, this is the first investigation about the phenolic composition, and antioxidant and antimicrobial potential of the *A. schurii* flowers extract, thus completing the literature data. The value of this research lies in its novelty, providing new scientific data concerning the polyphenolic compounds and the bioactivity of this species. In the ethanolic extract of *A. schurii*, 19 phenolic acids and flavonoids were screened by the HPLC-MS method. Nine of them—gentisic, caffeic, chlorogenic, *p*-coumaric acids, quercetin, and high amounts of isoquercitrin, rutin, luteolin and apigenin—were determined. Free radicals were directly detected in the extract with a pattern very similar to the pattern of polyphenols and phenolic acids extract composition, using EPR spectroscopy. According to the data obtained in this study, the extract was found to be a good natural antioxidant, possibly through an iron chelating mechanism, in different in vitro assays including DPPH bleaching, hemoglobin ascorbate peroxidase activity inhibition (HAPX) and inhibition of lipid peroxidation catalyzed by cytochrome c, methods related to the polyphenolic active principles content. Further studies will be needed to prove the mechanisms by which the compounds exert their antioxidant effects. In addition, the *A. schurii* extract showed significant antibacterial activity, and among the microorganisms, the most sensitive was *L. monocytogenes*. The first results of this study suggest the great value of *A. schurii* in terms of its possible pharmaceutical exploration.

## Figures and Tables

**Table 1:** Phenolic compounds in the *A. schurii* extract.

Compounds	[M − H]^−^, *m*/*z*	Retention Time (tR), min	Peak No.	UV Detection	MS Detection	Concentration (mg/100 g Plant Material)
Gentisic acid	153	3.69 ± 0.04	-	NO	YES	<0.02
Caffeic acid	179	6.52 ± 0.04	-	NO	YES	<0.02
Chlorogenic acid	353	6.43 ± 0.05	-	NO	YES	<0.02
*p*-Coumaric acid	163	9.48 ± 0.08	1	YES	YES	0.76 ± 0.11
Isoquercitrin	463	20.29 ± 0.10	2	YES	YES	68.75 ± 2.65
Rutin	609	20.76 ± 0.15	3	YES	YES	76.58 ± 3.42
Quercetin	301	27.55 ± 0.15	4	YES	YES	1.05 ± 0.24
Luteolin	285	29.64 ± 0.19	5	YES	YES	8.73 ± 0.96
Apigenin	279	39.45 ± 0.15	6	YES	YES	10.04 ± 1.06

Values are the mean ± SD (*n* = 3).

**Table 2:** Total content of polyphenols and antioxidant activity of the *A. schurii* extract.

Sample	TPC (mg GAE/g)	Flavonoids (mg RE/g)	Caffeic Acid Derivatives (mg CAE/g)	DPPH IC_50_ (µg·mL^−1^)	HAPX (mg RE/g)
*A. schurii*	96.93 ± 3.07	38.61 ± 2.39	41.48 ± 2.96	58.87 ± 2.12	1000 ± 129
Quercetin	-	-	-	5.47 ± 0.16	

Each value is the mean ± SD of three independent measurements. GAE: gallic acid equivalents; RE: rutin equivalents; CAE: caffeic acid equivalents; TPC: total polyphenols content; IC_50_: half maximal inhibitory concentration; DPPH: diphenylpicrylhydrazyl; HAPX: hemoglobin ascorbate peroxidase activity inhibition.

**Table 3:** Antimicrobial activity of the *A. schurii* extract.

Samples	Zone of Inhibition (mm)				
	*Staphylococcus aureus*	*Listeria monocytogenes*	*Escherichia coli*	*Salmonella typhimurium*	*Candida albicans*
*A. schurii*	16 ± 1.50	22 ± 1.00	11 ± 0.40	16 ± 0.10	6 ± 0.00
Gentamicin	19 ± 0.60	18 ± 1.00	22 ± 0.50	18 ± 0.00	-
Fluconazole	-	-	-	-	25 ± 0.20

Each value is the mean ± SD of four independent measurements. Gentamicin (10 µg/well) and Fluconazole (25 µg/well) were used as positive controls.
